# The trajectory of kinesiophobia in patients following total knee arthroplasty: a longitudinal qualitative study

**DOI:** 10.3389/fpsyg.2026.1776577

**Published:** 2026-02-11

**Authors:** Shuoni Ren, Yanan Kan, Lin Chen, Yijing Ling, Xiaolan Zhang, Wenjin Lv, Fuying Ye

**Affiliations:** 1 School of Nursing, Zhejiang Chinese Medical University, Hangzhou, Zhejiang, China; 2 Department of Orthopedics and Traumatology, The First Affiliated Hospital of Zhejiang Chinese Medical University, Hangzhou, Zhejiang, China; 3 Trade Union, The First Affiliated Hospital of Zhejiang Chinese Medical University, Hangzhou, Zhejiang, China; 4 Department of Nursing, The First Affiliated Hospital of Zhejiang Chinese Medical University, Hangzhou, Zhejiang, China

**Keywords:** kinesiophobia, longitudinal qualitative study, nurse, orthropaedic, total knee arthroplasty

## Abstract

**Aim:**

Based on Cognitive Dissonance Theory and the Lewin’s Change Model, this longitudinal study examines the developmental trajectory of exercise phobia in patients following total knee arthroplasty, aiming to provide evidence-based support for formulating rehabilitation strategies tailored to different stages.

**Method:**

To use purposive sampling, from December 2024 to June 2025, eight patients who underwent total knee arthroplasty at a Grade III Class A hospital in Zhejiang Province were selected as research subjects. To track the trajectory of kinesiophobia symptoms within 3 months post-surgery and complete data analysis with thematic analysis, we conducted a thematic analysis.

**Results:**

The trajectory of kinesiophobia in patients following total knee arthroplasty primarily manifests in two key aspects: cognitive assessment dysfunction (self-objectification and health literacy bias) and impaired behavioral control (pain central sensitization and rehabilitation motivation disorder).

**Conclusion:**

Following total knee arthroplasty, patients undergo a dynamic progression of kinesiophobia, characterized by a shift from cognitive appraisal imbalance to behavioral control impairment, driven by the persistent conflict between rehabilitation needs and fear-based cognition. To optimize postoperative quality of life, healthcare professionals should implement targeted interventions tailored to the phased characteristics of kinesiophobia.

## 1 Introduction

Knee osteoarthritis (KOA) is primarily characterized by joint pain and limited mobility (Pinto et al., 2017). The global aging population is accelerating, leading to a prevalence rate of degenerative knee joint disease as high as 10% (Brophy and Fillingham, 2022). Total knee arthroplasty (TKA) is often considered the optimal surgical procedure for treating end-stage KOA, significantly improving the function of the affected knee joint (Šťastný et al., 2016). Although early and sustained rehabilitation training is crucial for restoring range of motion by strengthening the muscles surrounding the joint, adherence to exercise rehabilitation among patients following TKA is generally low. Among these, kinesiophobia is a significant factor hindering the effective implementation of rehabilitation programs and delaying the process of muscle strength recovery (Cai et al., 2018). Kinesiophobia, also known as movement phobia (Luque-Suarez et al., 2019), refers to an excessive, irrational fear of physical activity caused by heightened sensitivity to movement due to painful stimuli. Studies indicate that the incidence of phobia in patients following TKA reaches as high as 50 to 70% (Luque-Suarez et al., 2019), and may potentially induce complications such as joint capsule adhesions and venous thrombosis (Filardo et al., 2016; Al-Amiry et al., 2022). Therefore, effectively controlling the onset of kinesiophobia is a crucial step in helping patients achieve positive rehabilitation outcomes.

Although cross-sectional studies using the Tampa Scale for Kinesiophobia (TSK-17) have confirmed a negative correlation between kinesiophobia severity and rehabilitation outcomes (Filardo et al., 2017; Padovan et al., 2018), such research fails to elucidate the psychological process whereby patients subjectively exaggerate and progressively entrench their kinesiophobia under the influence of immediate emotional disturbances such as pain and anxiety (Londhe et al., 2021b). Qualitative research indicates (Yanyan, 2019) that patients often overestimate their perceived risk associated with rehabilitation activities and exaggerate their kinesiophobia, leading them to adopt overly conservative rehabilitation plans. However, existing qualitative studies have primarily focused on specific time points such as peak pain periods, failing to fully reveal the dynamic changes in kinesiophobia that emerge throughout the rehabilitation process. Meanwhile, Tretter et al.’s findings, based on the TSK-17 scale changes, confirmed that the severity of kinesiophobia gradually decreased with rehabilitation progress (Tretter and Löffler-Stastka, 2024)^,^ exhibiting significant group-specific trends in its trajectory (Suc et al., 2009). However, these studies failed to delve into the intrinsic changes occurring at critical junctures of patient recovery.

This study integrates Cognitive Dissonance Theory (Tretter and Löffler-Stastka, 2024) and Lewin’s stages of change model (Suc et al., 2009) to construct an analytical framework. It posits that the psychological distress in patients with kinesiophobia arises from the conflict between catastrophic cognitions about functional exercise and rehabilitation needs. The resulting cognitive dissonance propels patients through three sequential stages of transformation: (1) a Cognitive Rigidity and Avoidance Phase, characterized by entrenched fear beliefs and systematic behavioral avoidance; (2) a Cognitive Triggering and Thawing Phase, marked by the dissolution of maladaptive cognitions and the loosening of avoidance patterns; and (3) a Cognitive Reconstruction and Attempt Phase, defined by the initiation of cognitive restructuring and attempts at new behavioral patterns. However, while “cognitive conflict theory” describes the process of cognitive and behavioral restructuring in phobic patients, it struggles to fully explain the underlying motivation driving these adaptive changes. According to Bandura (1977) “self-efficacy theory,” an individual’s belief in their ability to complete rehabilitation training is a key factor governing their behavioral choices and persistence. This theory elucidates how self-efficacy, as an intrinsic motivator, regulates patients’ cognitive assessments and behavioral control, thereby driving changes in their anxiety symptoms. In summary, this study integrates “Cognitive Dissonance Theory” and “Lewin’s Change Model” to construct an analytical framework and employs a longitudinal qualitative design, conducting semi-structured interviews with patients experiencing kinesiophobia following TKA at four distinct time points. This study aims to systematically delineate the stagewise progression of patients’ cognition and behavior regarding postoperative kinesiophobia, and to elucidate, based on self-efficacy theory, its pivotal role in driving this change process, thereby providing an evidence-based foundation for developing targeted intervention strategies.

## 2 Participants and methods

### 2.1 Participants and sampling

Patients with kinesiophobia after undergoing total knee arthroplasty (TKA) were recruited from the orthopedic center of a tertiary hospital in Zhejiang Province, China, using purposive sampling between December 2024 and June 2025. A purposive sampling approach based on the principle of maximum variation was employed. Participant selection was guided by key demographic characteristics (e.g., Age, educational level) and clinical disease features (e.g., preoperative health literacy). Inclusion criteria were: (1) diagnosis of knee osteoarthritis (KOA) and having undergone total knee arthroplasty (TKA), with no restriction on prosthesis type or fixation method; (2) assessed as having kinesiophobia at the first postoperative interview, defined as a score of ≥37 on the Tampa Scale for Kinesiophobia-17 (TSK-17) (Dupuis et al., 2023); (3) being alert, without cognitive or communication impairment, and able to understand and cooperate to complete the interviews; and (4) provision of informed consent and voluntary participation in the study. Exclusion criteria were: (1) history of or scheduled concurrent major joint replacement surgery (e.g., contralateral knee or ipsilateral hip); (2) comorbid conditions that could inherently cause kinesiophobia or severe functional impairment, such as severe neurological disorders or other inflammatory joint diseases; and (3) diagnosed severe psychiatric or cognitive disorders that could compromise understanding of the study or adherence to the longitudinal interviews. Withdrawal criteria were: (1) requirement for revision surgery during follow-up due to severe complications (e.g., rejection, periprosthetic infection) or major unforeseen changes to the rehabilitation plan; (2) voluntary withdrawal of informed consent and discontinuation of participation; (3) failure to complete longitudinal interviews at key timepoints (defined as ≤3 valid interviews), resulting in insufficient data for trajectory analysis. Sample size was determined according to the principle of thematic saturation in grounded theory (Hennink et al., 2019), with data collection concluded when no new themes emerged from the interviews and coding meaning reached saturation. A total of 8 patients with post-TKA phobia were recruited. Two participants were lost during the study: one declined to continue the interview at the third follow-up, and the other was lost to contact at the second follow-up due to an incorrect telephone number recorded in the hospital’s medical record system. The interview data collected from these two participants prior to attrition were retained and analyzed as supplementary contextual material. Notably, this study was designed to longitudinally describe the trajectory of kinesiophobia following TKA, rather than to compare pre- versus postoperative changes. Therefore, the TSK-17 score from the first postoperative assessment was used as an inclusion criterion, rather than a preoperative diagnosis of kinesiophobia.

### 2.2 Research methods

#### 2.2.1 Development of the interview guide

This study aims to investigate the cognitive and behavioral transformation process in patients with kinesiophobia following total knee arthroplasty (TKA) during the critical phase of functional recovery, particularly when their fear-related cognitions conflict with rehabilitation needs, and to analyze the pivotal role of self-efficacy as a core driving force in this process. Guided by the theoretical perspectives of Cognitive Dissonance Theory and Lewin’s Change Model, and integrating findings from the literature with clinical experience, the researchers developed an innovative semi-structured “Dual-Question Interview Framework.” The framework was designed to combine structured core questions with tailed probing, thereby facilitating an in-depth exploration of patients’ individualized conflict experiences and adaptive strategies. Additionally, taking into account the standard postoperative rehabilitation phases for TKA, the dynamic trajectory of kinesiophobia, and optimal clinical intervention windows, the researchers determined the follow-up time points at 1 week, 2 weeks, 1 month, and 3 months after surgery (Gilbey et al., 2003). Specifically, guidelines such as those from the American Academy of Orthopedic Surgeons (AAOS) recommend that patients begin rehabilitation exercises at 1 week after surgery, identifying this period as a critical window for transitioning from surgical trauma to active recovery (Brophy and Fillingham, 2022). Accordingly, this study set the “1-week postoperative” time point as the observation starting point for the longitudinal interviews. Moreover, Ziyan et al. (2022) indicated that the second week after TKA is not only a period during which the severity of kinesiophobia shows a slight increase, but also a critical phase for regaining the ability to perform activities of daily living (Min et al., 2015). Although functional recovery after total knee arthroplasty (TKA) is time-dependent, the optimal duration of postoperative rehabilitation programs remains a subject of ongoing debate. Filardo et al. (2016) noted that a one-month rehabilitation period is crucial for functional improvement, while kinesiophobia enters a critical transition phase from acute to chronic between 3 and 6 months after surgery. These two time points correspond to the critical period for early functional recovery and the intervention window for mid-to-long-term kinesiophobia, respectively, and were therefore also included as longitudinal follow-up points in this study. Based on pilot interviews conducted at four time points with one patient and a preliminary analysis of the resulting transcripts, this study integrated patient feedback with previous research findings to revise and refine the interview guide, ultimately producing the final version. The specific content was as follows: (1) Standardized core questions (applied consistently across all stages): “Are you currently concerned or afraid of performing rehabilitation exercises?” and “What makes you feel afraid?”, used to assess the experience of kinesiophobia symptoms in patients following TKA; (2) Individualized supplementary questions (stage-specific): “What form of assistance do you need most at the current stage?” (1 week postoperatively), “Could you describe your experience with rehabilitation therapy at this stage?” (2 weeks postoperatively), “What additional support is needed after discharge?” (1 month postoperatively), and “What form of health guidance do you require?” (3 months postoperatively).

#### 2.2.2 Data collection methods

Following the signing of the informed consent form, the researchers collected demographic and clinical characteristics (e.g., age, sex, preoperative health literacy level) from the patients’ electronic medical records. The initial semi-structured interview was conducted face-to-face at 1 week postoperatively, while the three subsequent qualitative follow-ups (at 2 weeks, 1 month, and 3 months after surgery) were completed via telephone to concurrently track patients’ recovery status, three quantitative measures were collected prior to each qualitative interview: (1) the Tampa Scale for Kinesiophobia-17 (TSK-17) was used to assess kinesiophobia symptoms—this scale was first administered during the first postoperative week for screening and enrollment, and was then repeated at each scheduled follow-up; (2) the Visual Analog Scale (VAS) was employed to evaluate pain intensity; and (3) the Oswestry Disability Index (ODI) was applied to measure functional outcomes. All interviews were conducted following a semi-structured guide, audio-recorded with participant consent, and lasted approximately 20–30 min each. Informational saturation served as the predetermined criterion for concluding the interviews. During the interviews, the researcher employed techniques such as in-depth probing and scenario simulation flexibly to engage with participants (Abildgaard et al., 2016), while simultaneously observing and recording nonverbal cues (e.g., tone of voice, facial expressions, and body reactions) to complement the audio-recorded data. The study employed ongoing participant recruitment and analysis until thematic stability was indicated by the data, at which point additional interviews were conducted until no new themes emerged, thereby verifying thematic saturation.

#### 2.2.3 Transcription and analysis methods

The data analysis in this study followed an inductive approach and was conducted within the Pattern-Oriented Longitudinal Analysis (POLA) framework (Kneck and Audulv, 2019). Specifically, the researchers adopted the constant comparative and iterative coding strategies within the qualitative content analysis approach outlined by Elo and Kyngäs (2008), allowing emerging insights to dynamically inform the focus and data collection in subsequent interviews. The audio recordings of each interview were transcribed independently by two researchers within 24 h post-interview, followed by repeated readings and cross-verification. The verified transcripts were then returned to the respective participants for content validation to confirm their accuracy. To facilitate longitudinal tracking and analysis, the data were systematically coded: participants were assigned unique identifiers in the order of enrollment (P1–P8), and the four interviews (at 1 week, 2 weeks, 1 month, and 3 months postoperatively) were labeled sequentially as A, B, C, and D. Each interview transcript was consistently formatted as “participant–interview phase” (e.g., P1-A). The coding process was conducted through three cyclical and iterative steps: (1) Initial coding: repeated reading of the text followed by line-by-line open coding to generate preliminary concepts; (2) Focused coding: constant comparison of codes across time points and participants to merge and cluster conceptually similar initial codes into preliminary categories; and (3) Axial coding: continuous comparative analysis of categories—examining their commonalities and differences—guided by the integrated framework of Cognitive Dissonance Theory and Lewin’s Change Model, leading to the iterative refinement of categories and sub-categories through addition, deletion, modification, or merging. To depict shifts in thematic emphasis across the trajectory of kinesiophobia, the researchers described and visualized the pattern of emergence for each theme by noting its relative frequency of mention across the four interview time points (T1–T4).

### 2.3 Ethical principles

This study adhered to the ethical principles outlined in the Declaration of Helsinki and was approved by the hospital’s ethics committee (ethical approval code: 2024-K-266-01). At the initial contact with potential participants, the researcher fully informed them about the study’s purpose, significance, procedures, and privacy protection measures. Written informed consent was obtained after they had achieved a thorough understanding. The consent form explained to participants that the study involved reviewing past clinical events, which might elicit emotions such as sorrow or distress, and it also sought their permission for the anonymized use and direct quotation of their responses in publications. To strictly protect patient privacy, all data were anonymized throughout processing by replacing personal information with unique identification codes, and only aggregated and de-identified summary data were used in the results. Given the longitudinal qualitative nature of this study, which can foster a unique trust-based relationship between the researcher and participants, the researcher closely monitored participants’ well-being and provided ongoing support as needed. Following the completion of data collection, the researcher clearly informed the participants of the conclusion of the research phase and, through appropriate communication, terminated the research relationship with care to prevent any sense of abandonment. To safeguard participant welfare, the corresponding author provided referral pathways to psychological support services for participants in need.

### 2.4 Rigor and reflexivity

The research team possessed extensive expertise in orthopedics and qualitative methodology, thereby ensuring methodological rigor throughout the study. Within the team, two researchers held a Master’s degree in nursing, and three had over 10 years of clinical nursing experience in orthopedics. This combined professional background ensured depth and breadth in data comprehension, analysis, and interpretation. Concurrently, this study adhered to a reflexive qualitative research methodology, emphasizing the continuous examination and clarification of the researchers’ prior experiences and potential biases to mitigate their undue influence on data interpretation. To ensure consistency in data collection, all interviews were conducted by the same qualified researcher and followed a unified semi-structured guide at four predetermined postoperative time points. Data analysis followed a rigorous process involving collaborative coding by multiple researchers and consensus building. First, at least two researchers independently performed initial coding and thematic extraction for each interview transcript. Next, the researchers jointly compared their analyses, thoroughly discussing similarities and differences in the coding. A third researcher was then invited to review the work. Any coding discrepancies were examined through iterative peer review between the researchers and the corresponding author until a consensus was reached. Finally, the resulting thematic framework and interpretations were submitted to the entire research team for collective deliberation and final approval.

## 3 Results

### 3.1 Participant characteristics

This study ultimately enrolled and completed longitudinal follow-up with eight patients exhibiting kinesiophobia following total knee arthroplasty (TKA), yielding a total of 34 valid interview transcripts (additional supplementary interviews were conducted for patients P4 and P7 due to interruptions during their scheduled sessions). To verify data saturation, three additional interviews were conducted with an additional participant; the analysis revealed no new themes, indicating that thematic saturation had been achieved. Detailed demographic characteristics and key clinical assessment metrics of the study participants are presented in Tables 1, 2.

**Table 1:** Demographic and clinical characteristics of the participants (*n* = 8).

Characteristic	Category	Number
Gender	Male	6
	Female	2
Age (years)	≤ 65 years	3
	> 65 years	5
Marital status	Married	7
	Unmarried	1
Educational level	Elementary school	5
	Junior high school or above	3
Surgical approach (years)	≤8 years	5
	>8 years	3
Surgical	Robot assisted	2
	Non-robot assisted	6
Rehabilitation destination	Home-based rehabilitation	5
	Rehabilitation hospital	

**Table 2:** Changes in selected clinical measures across postoperative time points (mean ± sd).

Scale Scoring	1 week	2 week	1 month	3 months
Tremor Severity Scale (TSK-17)	52.4 ± 5.8	48.1 ± 6.3	41.6 ± 7.5	36.8 ± 8.2
Pain Intensity (VAS Scale)	7.5 ± 1.2	5.2 ± 1.5	3.4 ± 1.8	2.1 ± 2.0
Functional Disability Level (ODI Scale)	72.5 ± 6.8	58.2 ± 8.5	38.4 ± 10.2	26.8 ± 12.5

Values are mean ± SD ODI percentage.

**Figure 1:**
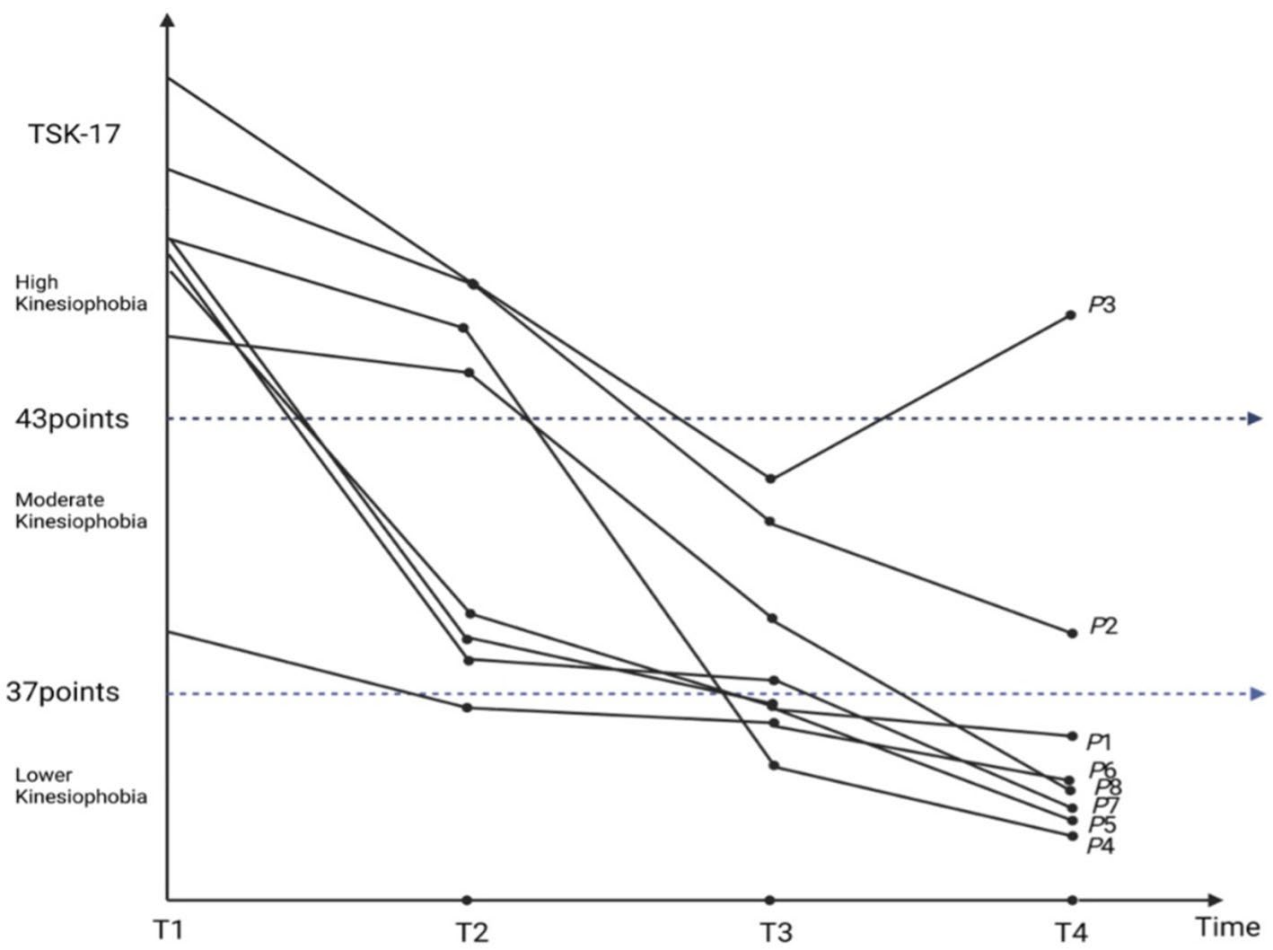
Trajectory of kinesiophobia in patients following TKA.

**Figure 2:**
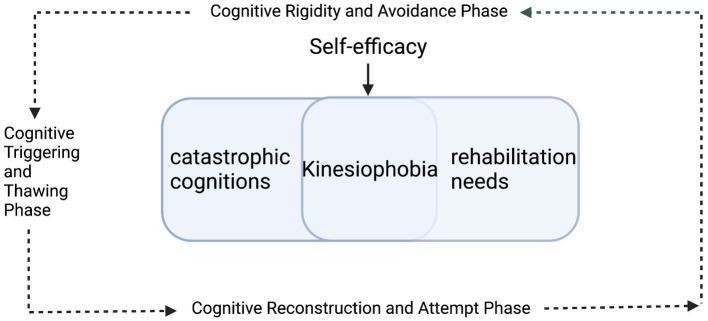
A framework for kinesiophobia trajectory following total knee arthroplasty (TKA) integrating cognitive dissonance theory and Lewin’s change model.

Drawing on longitudinal kinesiophobia trajectories from eight TKA patients (Figure 1), and guided by the integrated Cognitive Dissonance Theory and Lewin’s Change Model framework (Figure 2), this study illustrates how self-efficacy shapes the progression from cognitive-appraisal dysregulation to deficits in behavioral control. In this framework, “cognitive appraisal dysregulation” is primarily manifested as self-objectification and health literacy biases, while “behavioral control impairment” is closely associated with pain central sensitization and rehabilitation behavior avoidance. This dynamic process is delineated into three sequential phases: the Cognitive Rigidity and Avoidance Phase, the Cognitive Triggering and Thawing Phase, and the Cognitive Reconstruction and Attempt Phase. Self-efficacy serves as a core moderating variable that influences how patients respond to “cognitive appraisal dysregulation” and “behavioral control impairment,” thereby modulating the trajectory of kinesiophobia symptoms. Higher self-efficacy buffers cognitive dissonance and facilitates adaptive behavioral engagement. In contrast, lower self-efficacy intensifies cognitive dissonance and reinforces behavioral avoidance, thereby acting as a perpetuating factor for persistent or worsening kinesiophobia.

## 4 Theme 1: cognitive appraisal dysregulation

### 4.1 Subtheme 1.1: self-objectification

Lower levels of self-efficacy were significantly associated with a propensity for self-objectification within caregiver-patient interactions. This passive role identity was manifested as absolute compliance with external directives, coupled with reduced rehabilitation autonomy, thereby exacerbating the fear of functional exercise. However, as patients’ adherence to these passive role identities decreased, their perceived autonomy regarding rehabilitation increased significantly.

(1) Cognitive Rigidity and Avoidance Phase: surgical trauma and other external stimuli were closely linked to patients’ early postoperative avoidance of functional exercise. This behavioral avoidance fostered an experience of diminished bodily control and was subsequently associated with the emergence of kinesiophobia symptoms. During this phase, reduced trust in bodily perception was commonly reported, alongside a pronounced reliance on external support for rehabilitation decisions. This dependency was evidenced by patients transferring decision-making authority to family members, exemplified by statements such as, “I listen to my children—whatever they think is good, is good for me” *(P6-A)*, or explicitly noting, “I refused the therapist’s intervention until my daughter made the decision” *(P6-A)*. (2) Cognitive Triggering and Thawing Phase: patients with lower self-efficacy frequently reported uncertainty in perceiving knee joint range of motion, which compromised their ability to distinguish between target and safe activity thresholds during rehabilitation. This perceptual uncertainty often led patients to attribute physical limitations to immutable physiological factors. For example, one patient ascribed their constraints to age: “I know my own body—now that I’m older, my joints and muscles just don’t work as they used to” *(P4-B)*. In contrast, patients with higher self-efficacy exhibited more proactive engagement in decision-making, including actively seeking information, participating in program discussions, and taking the lead in implementation, as one participant stated, “I feel I can’t just shirk all responsibility” *(P7-A)*. (3) Cognitive Reconstruction and Attempt Phase: with enhanced self-efficacy, some patients demonstrated a greater sense of responsibility toward rehabilitation decision-making. An observable linguistic shift was noted, in which patients’ references to the primary decision-maker transitioned from “they” to “I” or “myself” when discussing rehabilitation-related choices. Patients articulated their role in the rehabilitation process more proactively and explicitly stated their intention to participate in decision-making. For instance, one patient noted, “I know the postoperative period is the golden window for recovery. If you miss this phase, the effectiveness of later exercises will be significantly reduced” *(P6-C)*.

### 4.2 Subtheme 1.2: health literacy bias

During clinician-patient communications, individuals with limited health literacy commonly revealed a gap between their personal beliefs and the professional recommendations received, concurrently reporting low self-efficacy in managing their rehabilitation activities. During the rehabilitation process, patients often exhibited hesitation in decision-making and actively postponed or avoided specific activities they perceived as risky. In contrast, patients who received targeted postoperative education demonstrated less repeated questioning or verification of information when discussing rehabilitation risks and conveyed their confidence in recovery in more direct and assured terms. During the later phase of rehabilitation, patients with limited health literacy diverged into two distinct patterns of adherence behavior: some exhibited selective compliance with medical advice, accepting only those recommendations that aligned with their personal understanding or preferences; while others demonstrated full adherence to clinical instructions, expressing alignment with the traditional clinician–patient role structure and attributing decision-making responsibility entirely to healthcare providers.

(1) Cognitive Rigidity and Avoidance Phase: patients with lower health literacy demonstrated a tendency to catastrophize somatic sensations experienced during rehabilitation. This cognitive pattern was associated with both decreased adherence to essential functional exercises and reduced confidence in performing them. For example, one patient stated, “*Whenever I feel that aching or swelling sensation in my knee, I worry that it might be bleeding again, and I don’t dare move it at all” (P2-A)*. In contrast, some patients with higher health literacy, while explicitly stating their intention to exercise, demonstrated a cognitive bias in processing risk information. This bias was characterized by selective attention to and amplification of negative information that resonated with their personal fears. This cognitive tendency manifested in their rehabilitation practices as observable hesitation and conservative behavior. For instance, one patient expressed this conflict: “I read online that you shouldn’t get out of bed too early, but my doctor always says I can. Whose advice should I follow?” *(P3-A)*. (2) Cognitive Triggering and Thawing Phase: the influence of social interaction on patients’ rehabilitation cognition and behavior varied with their health literacy levels. Among patients with lower health literacy, positive social interactions often served as a key channel for obtaining practical rehabilitation experience and emotional support. Such interactions facilitated the initial development of patients’ self-management awareness regarding functional exercises, manifested through learning specific activity techniques or gaining motivational support via peer communication. The statement from one participant clearly illustrates this linkage between risk perception and behavioral caution: “I believe I should wait a while longer, because I have learned from social media and other patients that the risk of joint dislocation at this stage is particularly high” *(P8-C)*. (3) Cognitive Reconstruction and Attempt Phase: during the later stages of rehabilitation, patients diverged in their reliance on medical authority. One subgroup demonstrated full adherence to clinical instructions and actively deferred decision-making authority to healthcare providers. One subgroup expressed full trust in the medical system, as captured in the statement, “I consider this hospital excellent and completely trust the doctors’ professional judgment” *(P4-D).* In contrast, another subgroup verbally affirmed the authority of healthcare providers while exhibiting selective adherence in practice. Their compliance was primarily limited to recommendations concerning activity restriction. Decisions related to engaging in functional exercises, however, were often based on personal interpretation of somatic sensations. This was explained by one participant: “I believe most of what the doctor says is correct, but I also need to adjust based on my actual condition” *(P3-C)*.

## 5 Theme 2: impaired behavioral control

### 5.1 Subtheme 2.1: pain central sensitization

Patients’ perception of postoperative pain intensity was closely linked to their self-reported confidence in functional exercise and fear of activity. Multiple patients with a long-term history of knee pain described that persistent or sudden pain after total knee arthroplasty (TKA) often rapidly triggered concerns about prosthesis loosening or surgical failure. This then manifested as active restrictions in the intensity or range of their movements. Notably, even among patients with high health literacy, intense pain was found to exacerbate their concerns regarding rehabilitation risks, resulting in overly cautious or avoidant behaviors during exercise. However, when patients received systematic pain management and achieved symptom relief along with functional improvement, their fear of pain shifted to a focus on functional progress. This shift was accompanied by a self-reported reduction in fear of activity.

(1) Cognitive Rigidity and Avoidance Phase: patients commonly reported persistent discomfort such as incisional sharp pain after TKA and demonstrated pathological vigilance towards these painful sensations. Some patients indicated that this pain experience reactivated past traumatic memories, leading them to interpret the pain as a definitive signal of re-injury directly. The fear of exacerbating pain or causing re-injury led to a distinct avoidance behavior pattern. This included active avoidance of large-range joint motions, refusal or reduction of self-perceived weight-bearing exercises, and evident initial hesitation with frequent movement pauses during activity attempts. This apprehension was vividly captured in one participant’s account: “Even lying down hurts. Is there any way to lessen this pain? I am terrified that moving will cause another injury” *(P2-A)*. (2) Cognitive Triggering and Thawing Phase: patients who held an expectation of a complete cure from TKA were more prone to engage in prospective risk assessment, driven by concerns that activity might induce pain and compromise joint function. Guided by their personal interpretation of pain and exposure to risk-related information, these patients tended to establish clear safety boundaries for themselves. This manifested as increased reliance on caregivers or assistive devices for activities they could otherwise perform independently, and the deliberate maintenance of what they perceived as an “ideal” or “safe” posture. This mindset was succinctly captured by one participant: “If I damage the prosthesis because of my own activity, all the money and effort I invested would be wasted” *(P1-C)*. (3) Cognitive Reconstruction and Attempt Phase: after receiving interventions including pain neuroscience education and physical therapy, patients reported an increased sense of pain controllability and a greater acceptance of pain as a normal experience. They came to view pain as part of the recovery process. With enhanced self-efficacy, patients began to attempt activities within a personally acceptable range of pain. Although this process still involved a certain degree of pain, the level of agreement within this patient group regarding the role of rehabilitation training in alleviating kinesiophobia increased correspondingly. This cognitive normalization is illustrated by one participant’s reflection: “It does hurt [hesitates], but that’s completely normal. To be honest, it would be unreasonable to feel no pain at all after surgery” *(D5)*.

### 5.2 Subtheme 2.2: rehabilitation motivation disorder

The degree to which rehabilitation motivation was internalized emerged as a primary determinant of patients’ cognitive-behavioral coping styles. This process of internalization was often mediated by whether the family support they received was autonomy-supportive and motivational in nature. For some solitary elderly patients following total knee arthroplasty (TKA), family support that primarily provided daily care while lacking encouragement for autonomy did not correspond with a marked improvement in their rehabilitation self-efficacy. When patients internalized their rehabilitation motivation into a sense of personal responsibility, their self-efficacy for functional exercise was typically higher. This enhanced self-efficacy often drove a shift in patients’ rehabilitation decisions from passive avoidance to active coping, accompanied by a reduction in kinesiophobia severity.

(1) Cognitive Rigidity and Avoidance Phase: when family assistance primarily consisted of companionship and caregiving support, it did not translate into enhanced autonomy or self-efficacy in the patient’s rehabilitation process. This deficiency in intrinsic motivation inclined patients toward adopting negative and avoidant behavioral strategies during rehabilitation, making the continuation of kinesiophobia behavior patterns more likely. As one participant described this conflict: “It’s mainly at the moment when I need to bend my knee that I feel anxious, but I also know that not moving at all will hinder recovery even more. That’s why I need my family now” *(P7-A)*. (2) Cognitive Triggering and Thawing Phase: for some elderly patients, a positive attitude toward rehabilitation stemmed from a profound desire to swiftly regain self-care ability and alleviate the burden on their family. This desire led to a shift in the patients’ cognitive focus from a fear of immediate pain to a concern for long-term joint function and quality of life. Furthermore, the patients’ sense of responsibility and dedication to their families served to significantly strengthen their willingness to confront fear and take action. This is exemplified by one participant’s account: “It was precisely because of the fear that I became more proactive in consulting my doctors, and they explained the root cause behind my anxiety” *(PB-7)*. (3) Cognitive Reconstruction and Attempt Phase: following psychological interventions such as cognitive behavioral therapy, some patients underwent a deeper motivational shift. They transformed the task of adhering to functional exercises from a passive obligation into an active, personal responsibility for their own rehabilitation process. This sense of responsibility propelled them to begin setting specific, actionable personal rehabilitation goals and articulated an expectation for improved postoperative quality of life. As one participant stated: “I have now set daily goals, firmly believing that consistent exercise will improve my pain and gait” *(PD-7)*.

## 6 Discussion

Guided by an integrated framework of Cognitive Dissonance Theory and Lewin’s Change Model, this study pioneered a longitudinal qualitative approach to trace the progression of kinesiophobia following total knee arthroplasty (TKA). By elucidating the frequency dynamics of core themes across rehabilitation stages, it systematically reveals the underlying cognitive and behavioral shifts that characterize the evolution of kinesiophobia. In the T1 phase, avoidance and hesitation toward rehabilitation were primarily driven by central sensitization to pain, while deficits in health literacy further amplified fear by reinforcing catastrophic interpretations of pain. During the T2 phase, excessive worry about the risks of functional exercise served as a cognitive signature of a passive “self-objectification” state. Notably, a “health literacy bias” at the cognitive level persisted, manifesting specifically as inconsistent understanding of, or lack of confidence in, the key techniques of rehabilitation exercises. As rehabilitation progressed into the T3 phase, “impaired behavioral control” was gradually restored and “self-objectification” attenuated in some patients. However, the persistent “health literacy bias” accounted for significant inter-individual differences in the rate and extent of kinesiophobia alleviation. In the T4 phase, the onset of rehabilitation motivation disorders precipitated a marked divergence in the degree of kinesiophobia alleviation. More importantly, health literacy bias exerted its most prominent effect at this stage, ultimately emerging as the critical determinant of long-term rehabilitation outcomes following TKA.

Patients in this study reported notable pain in the immediate postoperative period, aligning with a primary focus of early clinical management. Despite objective improvements from robotic-assisted TKA (Fu et al., 2024), and effective pain control via multimodal analgesia (Nambi et al., 2023) and exercise therapy (Booth et al., 2017), our qualitative analysis revealed a critical divergence: for some patients, recovery was dominated by a disproportionately intense, pain-related fear that persisted despite diminishing nociceptive input. This suggests a decoupling within the typical fear-pain dyad, where fear becomes a self-sustaining driver of the pain experience. This indicates a fundamental reconfiguration in the pain experience, where its core shifts from sensory intensity to a perceptual distortion mediated by central nervous system remodeling (Swinkels-Meewisse et al., 2003). This reconfiguration means that pain transforms into a mind–body state caught in a self-perpetuating cycle, driven and maintained by anticipatory anxiety (Trocoli and Botelho, 2016; Xie et al., 2024). Therefore, this study posits that effective postoperative pain management must extend beyond the optimization of analgesic regimens to incorporate deliberate cognitive restructuring, thereby fostering the patient’s role as an active agent and enhancing their self-efficacy in the pain management process. A forward-looking application involves developing the digital pain mapping technique by Minetto et al. (2025) into a standardized pain feedback tool. This approach addresses a fundamental gap in conventional pain assessment by quantifying spatial distribution, while its greater utility lies in translating subjective symptoms into an objective visual map. This visualization provides patients with an intuitive means to track the dynamic changes in pain location and intensity, thereby introducing a new dimension to personalized monitoring and management. Furthermore, we endorse the initiative proposed by Thomas for systematically addressing kinesiophobia within preoperative prehabilitation programs (Bandholm et al., 2025). The core of this approach involves facilitating a cognitive shift from a threat-focused appraisal of pain to an adaptive belief in the therapeutic benefit of safe, guided rehabilitation exercises. This cognitive restructuring, by promoting the understanding that movement is conducive to pain relief and functional recovery, is essential for reducing fear-driven avoidance behaviors and disrupting the fear-pain cycle.

The study revealed that some patients exhibited high compliance with external instructions alongside a marked absence of engagement in the decision-making process. This finding suggests that interventions during the second postoperative week should aim to guide patients from being passive recipients of care towards becoming active co-designers of their rehabilitation. This observation is in line with the concept advocated by Elwyn et al. (2012), which emphasizes promoting decision-making participation by helping patients explore their preferences. Therefore, our orthopedic center developed a multidisciplinary intervention model centered on empowerment and collaboration: physicians were responsible for providing authoritative explanations to reshape patients’ illness perceptions; physiotherapists constructed successful experiences through safe demonstrations; and nurses were tasked with continuously reinforcing positive behaviors and providing feedback. This model was designed based on self-efficacy theory, integrating the patient self-feedback mechanism advocated by Phelps et al. (2019) into the concrete steps and progression of the tiered exercise protocol. This approach directly addresses the practical challenge of patients’ lack of decisional engagement identified in the literature (Ottenvall Hammar et al., 2014). Furthermore, our study revealed that caregivers tended to adopt risk-averse, defensive caregiving strategies, often substituting their own perceptions and judgments for those of the patient regarding pain and decision-making—an observation consistent with the findings of Shin and Lee (2018). This pattern of substitution can readily exacerbate the patient’s deficit in decisional engagement and may foster doubts about their own self-judgment. Therefore, future interventions should prioritize facilitating a role transition for caregivers—from making substitutive decisions to supporting patient autonomy. This requires training in effective communication that empowers patients in their own care choices. When caregivers are skilled at recognizing patients’ specific progress and providing consistent emotional support with positive feedback, it will aid in gradually enhancing patients’ autonomy in rehabilitation decisions and their engagement with prescribed exercises.

As joint function gradually recovers 1 month after TKA, the patient’s need for autonomous control over rehabilitation activities correspondingly increases. Therefore, the focus of intervention at this stage should shift from maintaining basic function to establishing a rehabilitation support system that is centered on enhancing self-efficacy, with the central aim of strengthening patients’ sense of control over the recovery process through an effective self-monitoring framework. This direction is supported by existing evidence, as demonstrated by Ottenvall Hammar et al. (2014), whose study showed that using an activity diary significantly improves functional exercise outcomes in patients after TKA. It is therefore recommended to introduce a visual progress-tracking tool for parameters such as joint range of motion and exercise duration, with reference to protocols like that of Ottenvall Hammar et al. (2014), which utilized visual monitoring within a targeted resistance training program to strengthen the operated-side muscles. Alongside this, it is pertinent to explore an integrated telerehabilitation model supported by smart technologies, for instance, by incorporating wearable sensors to enable real-time monitoring of kinematic data including gait and joint range of motion (Nuevo et al., 2024). This integrated strategy not only provides patients with real-time awareness and a sense of control over their health status but also addresses their intrinsic need for autonomy and flexibility during rehabilitation (Kaambwa et al., 2015). However, some patients with kinesiophobia still struggle to reconcile rehabilitation autonomy with the urge to avoid discomfort, a conflict often manifested by frequently seeking reassurance from healthcare providers to validate the normality of their recovery progress (Larsson et al., 2022). Grounded in self-efficacy theory, sharing success stories of peers represents a viable strategy for enhancing patients’ cognitive appraisal and self-management capabilities. This approach centers on integrating symptom-normalizing information into narrative accounts (Andersen et al., 2025), by guiding patients to recognize fatigue and emotional fluctuations as common recovery experiences and facilitating vicarious learning of effective coping strategies for postoperative discomfort. A notable, and somewhat unexpected, finding was that the majority of participants reported the act of sharing their own experiences facilitated a reframing of the meaning of their negative events. Particularly when coupled with the awareness that their sharing could benefit others, this narrative process itself generated a positive, inherent therapeutic value.

The findings of this study indicate that the three-month period following surgery constitutes a pivotal period during which rehabilitation motivation undergoes a critical shift, significantly driving engagement in functional exercise behaviors. This insight not only aligns with Bandura’s theory positing self-efficacy as a core driver of behavioral persistence (Bandura, 1977), but also clarifies that the variation in patient adherence is essentially mediated by their differential capacity to internalize self-efficacy into a stable form of rehabilitative autonomy. Consequently, this evidence, combined with prior research (Chou et al., 2016), collectively positions self-efficacy not merely as an initial catalyst, but as the central sustaining force that underpins the maintenance of the rehabilitation process in its later stages. Therefore, clinical practice must advance beyond behavioral supervision to actively facilitate the deep transformation and sustained self-regulation of rehabilitation motivation—cultivating the intrinsic driver necessary for long-term maintenance. This necessitates implementing a continuous, structured support system, responsive to patients’ stage-specific needs, to systematically compensate for limitations in self-management capacity. Such a support system directly addresses the phased and intensified rehabilitation guidance that patients consistently expect, as identified by Knoop et al. (2022), and is fundamentally aligned with the core rehabilitative value of regaining independence (Hladkowicz et al., 2023). As a preliminary exploration in this direction, we propose a nursing-led integrated support model. This model encompasses regularly scheduled structured rehabilitation workshops, the establishment of peer-supported follow-up groups, and the utilization of digital platforms by designated nurses to provide remote consultations and home-based rehabilitation guidance, while also sustaining interactive online patient communities. The primary objective of this model is to establish an accessible, structured framework of external support and supervision for discharged patients, designed to empower them in proactively.

Through an in-depth analysis of eight patients with kinesiophobia following TKA who varied in key baseline characteristics, this study sought to understand how background factors such as age and educational level shape their experiences of and responses to symptoms of “cognitive appraisal dysregulation” and “impaired behavioral control.” For some older patients with lower educational attainment, their narratives were deeply embedded in traditional beliefs such as “it takes a hundred days for muscles and bones to heal.” This perspective was intertwined with a more intense perception of pain and the setting of conservative rehabilitation goals (Aily et al., 2021) a finding consistent with the report by Xu Huiping et al. (2021). Younger patients with higher levels of education, however, achieve cognitive restructuring and engage in more proactive self-regulation attempts at an earlier stage. Building on these insights, stratified cognitive-behavioral interventions can be developed. For vulnerable subgroups such as older adults with lower educational attainment, intervention design must prioritize accessibility and usability. As Zhu et al. (2023) have confirmed, there is a significant need for and acceptance of user-friendly digital health services among older adults in China. Therefore, implementing senior-friendly adaptations to mobile health applications—for instance, by enlarging font sizes, simplifying operational procedures, and incorporating voice-assisted interactions (Nuevo et al., 2024) is warranted. These adaptations can lower the technological barrier, mitigate patients’ perceptions of rehabilitation as overly complex, and thereby facilitate the translation of rehabilitation intention into sustained behavior. Particularly for older TKA patients with concurrent hearing or speech impairments, the rehabilitation challenge deepens beyond technical and cognitive domains. Functional limitations frequently prompt existential distress centered on the “fragility of life.” Therefore, perioperative care should extend beyond standard pain management and assistive device guidance (Londhe et al., 2021a) to integrate psychologically informed support, led by counselors or trained caregivers. This support should aim to facilitate a reconstruction of the patient’s sense of self-worth and meaning in life. It is noteworthy that while younger patients often possess better physical conditioning and a more informed understanding of recovery, their rehabilitation motivation can be complicated by an excessive preoccupation with meeting the expectations of others. This finding suggests that clinical interventions for this group should refocus their efforts towards facilitating reintegration into meaningful social roles, thereby alleviating the anxiety and helplessness stemming from difficulties in role adaptation. This perspective is supported by Nambi et al. (2023), who posit that social role identification can serve as a key mechanism for enhancing self-efficacy, which in turn significantly improves rehabilitation outcomes and mitigates kinesiophobia symptoms. Therefore, when guiding younger patients to establish realistic rehabilitation expectations, clinicians should deliberately link functional recovery to tangible, role-specific objectives, such as reintegrating into family life, returning to work, and resuming social activities. While this exploratory analysis offers initial insights into the experiential patterns among patients with diverse backgrounds, the limited sample size necessitates further validation in larger-scale studies. Future research could systematically examine the moderating effects of these factors within quantitative designs and deepen the understanding of the heterogeneous mechanisms underlying kinesiophobia by employing structured mixed-methods approaches. Nonetheless, the present findings provide valuable preliminary clues for comprehending the complex manifestations of kinesiophobia after TKA and for informing the development of personalized intervention strategies.

Based on an in-depth analysis of the interview data, this study found within its limited sample that initial disparities in health literacy emerged as a pivotal factor shaping the cognitive and behavioral responses to kinesiophobia post-TKA. Furthermore, the analysis suggests a dynamic interplay between the level of health literacy and processes of pain central sensitization (Burns et al., 2015; Riddle et al., 2010), which together appear to influence patients’ adherence to rehabilitation exercises (Mancuso et al., 2008; Hamilton et al., 2013). These dynamics manifested through four distinct patterns. First, during periods of high pain intensity, patients—regardless of health literacy level—were universally observed to enter a phase of Cognitive Rigidity and Avoidance. Second, patients with high health literacy, upon experiencing a reduction in pain, proactively integrated information and professional guidance. This enabled them to transition into the Cognitive Triggering and Thawing Phase (Goudie et al., 2018) and subsequently achieve the Cognitive Reconstruction and Attempt Phase. Third, in contrast, patients with lower health literacy often remained entrenched in maladaptive beliefs. Even with pain alleviation, they typically required external intervention to move from a state of potential Cognitive Triggering toward active Cognitive Reconstruction and Attempt. Finally, a fourth pattern was theoretically posited but less frequently encountered in our interviews: when pain levels were very low and health literacy was high, no significant conflict arose between the two factors. This pattern was encountered less frequently in our interviews, a relative scarcity that likely stems from the study’s focus on the early postoperative period dominated by pain-induced central sensitization. Importantly, these identified patterns are not static; they evolve dynamically throughout the rehabilitation process, thereby exerting a significant influence on long-term outcomes. This influence is concretely reflected in the divergent recovery pathways patients subsequently choose, with some transitioning to home-based rehabilitation upon symptom relief, while others continue to seek structured care in professional facilities. Zelle et al. (2016) note that when patients perceive necessary physical activity as a health threat, the resulting kinesiophobia frequently undermines rehabilitation adherence. This underscores the necessity for implementing tailored, phased interventions that are responsive to the patient’s stage-specific cognitive and behavioral presentation, particularly in relation to the dynamics of pain central sensitization across the postoperative recovery timeline to address this need in the T1 phase, our orthopedic center developed a visual patient education handbook tailored for patients with lower health literacy. It consolidates core secondary prevention information on post-TKA kinesiophobia management and exercise protocols. Preliminary application suggests that this handbook helps bridge the cognitive gap between patients’ subjective discomfort and objective clinical indicators (Partridge et al., 2012; Dahlberg et al., 2018), thereby encouraging them to reframe pain as a manageable challenge rather than an absolute threat to be avoided (Ahlstrand et al., 2017). During the T2 stage, some patients exhibit selective trust in medical authority (Murray and Parkinson, 2018; Jourdan et al., 2012), tending to adopt medical advice that aligns with their pre-existing expectations while still passively adhering to traditional physician-patient roles (Wong et al., 2015). Clinical interventions may actively leverage patients’ existing trust in medical authority by embedding health education into patients’ existing information frameworks (Pendlimari et al., 2012). For instance, providing systematic perioperative health education to patient P7 enhanced his health literacy, which subsequently led to more prudent medical decision-making and contributed to a reduction in both readmission rates and unplanned healthcare service utilization (Lihong, 2023). In the T3 stage, in response to the needs of patients with high health literacy for autonomous health information management, intelligent home-based rehabilitation systems centered on embedded wearable sensors and machine learning algorithms are driving a paradigm shift in rehabilitation care (Ramkumar et al., 2019). These systems hold promise for delivering personalized feedback while significantly reducing reliance on traditional high-frequency, face-to-face consultations. During the T4 stage of later rehabilitation, the provision of personalized digital resources through social media interaction promotes the progression of rehabilitation behaviors toward a more proactive and refined phase of self-regulation. Bandura’s self-efficacy theory posits that self-efficacy shapes behavioral expectations and standards within a social framework (Bandura, 1977). The interactivity and supportiveness of the digital environment can be leveraged to assist patients in reconstructing and practicing the positive social roles they aspire to, thereby facilitating a fundamental shift from passive acceptance to active self-management. Therefore, by implementing a differentiated intervention pathway that combines universal guidance in the early stages with targeted reinforcement in later phases, it is possible to systematically address disparities in health literacy among post-TKA patients and facilitate the transformation of both cognitions and behaviors related to kinesiophobia.

### 6.1 Strengths and limitations

A key strength of this study lies in its innovative integration of dual theoretical perspectives—“cognitive conflict” and “self-efficacy”—to elucidate the dynamic process of kinesiophobia across cognitive and behavioral dimensions, along with its underlying psychological drivers. As a longitudinal qualitative inquiry designed to explore this process in depth rather than to test variable associations, the modest sample size, while permitting only general demographic reporting, is fully aligned with the interpretive aims of the research. Furthermore, methodological rigor was enhanced through the consistent maintenance of a reflexive journal during data analysis, to ensure transparency and to mitigate the potential influence of researcher positionality. Several limitations should be acknowledged. First, given the exploratory purpose and sample size of this qualitative study, it was not designed to systematically analyze the specific effects of factors such as health literacy and self-efficacy on the lived experience of kinesiophobia. Addressing this question would require a different research design, such as a mixed-methods approach. Second, the single-center design, along with the influence of local clinical culture, treatment protocols, and patient demographics, may constrain the transferability of the identified kinesiophobia trajectory to other settings. Finally, by focusing primarily on the early postoperative phase where symptom fluctuation is most pronounced, the study may not fully capture the longer-term evolution of kinesiophobia or its relationship with ultimate functional outcomes. Finally, the potential influence of the researcher-participant dynamic on the data generation process cannot be entirely discounted. Building on these limitations, we propose that future research employ multicenter longitudinal designs to validate and extend the kinesiophobia trajectory identified here, with particular attention to moderating contextual factors such as healthcare accessibility. Extending the follow-up period will also be crucial to elucidate the long-term course of kinesiophobia and its definitive impact on functional recovery outcomes.

## 7 Conclusion

This longitudinal qualitative study demonstrates that the severity of kinesiophobia following total knee arthroplasty follows a dynamic trajectory, shaped significantly by the patient’s sense of self-efficacy. This process involves two core dimensions: “cognitive appraisal dysfunction,” manifested as self-objectification and health literacy bias; and “impaired behavioral control,” manifested as pain central sensitization and disordered rehabilitation motivation. This study identified that health literacy disparities, associated with factors such as age and education level, serve as a stable cognitive factor continuously influencing the trajectory of kinesiophobia, with their impact becoming more pronounced in later stages. Therefore, we propose that future research should design and implement targeted stepped psychological-behavioral intervention protocols within multicenter orthopedic settings, based on the staged evolution patterns of cognitive and behavioral symptoms identified here, to evaluate their effectiveness in improving long-term rehabilitation outcomes for patients following different kinesiophobia trajectories.

## Data Availability

The original contributions presented in the study are included in the article/supplementary material, further inquiries can be directed to the corresponding author.

## References

[ref1] Abildgaard J. S. Saksvik P. Nielsen K. (2016). How to measure the intervention process? An assessment of qualitative and quantitative approaches to data collection in the process evaluation of organizational interventions. Front. Psychol. 7:1380. doi: 10.3389/fpsyg.2016.01380, 27713707 PMC5031711

[ref2] Ahlstrand I. Vaz S. Falkmer T. Thyberg I. Björk M. (2017). Self-efficacy and pain acceptance as mediators of the relationship between pain and performance of valued life activities in women and men with rheumatoid arthritis. Clin. Rehabil. 31, 824–834. doi: 10.1177/0269215516646166, 27146888

[ref3] Aily J. B. de Almeida A. C. Ramírez P. C. da Silva Alexandre T. Mattiello S. M. (2021). Lower education is an associated factor with the combination of pain catastrophizing and kinesiophobia in patients with knee osteoarthritis? Clin. Rheumatol. 40, 2361–2367. doi: 10.1007/s10067-020-05518-1, 33230685

[ref4] Al-Amiry B. Rahim A. Knutsson B. Mattisson L. Sayed-Noor A. (2022). Kinesiophobia and its association with functional outcome and quality of life 6-8 years after total hip arthroplasty. Acta Orthop. Traumatol. Turc. 56, 252–255. doi: 10.5152/j.aott.2022.21318, 35968616 PMC9612671

[ref5] Andersen T. C. Wilhelmsen M. Lian O. S. (2025). 'The MRI-scan says it is completely normal': reassurance attempts in clinical encounters among patients with chronic musculoskeletal pain. Health (London) 29, 489–509. doi: 10.1177/13634593241290185, 39428652 PMC12235064

[ref6] Bandholm T. Husted R. S. Troelsen A. Thorborg K. (2025). Changing the narrative for exercise-based prehabilitation: evidence-informed and shared decision making when discussing the need for a total knee arthroplasty with patients. Osteoarthr. Cartil. Open 7:100601. doi: 10.1016/j.ocarto.2025.100601, 40170680 PMC11960630

[ref7] Bandura A. (1977). Self-efficacy: toward a unifying theory of behavioral change. Psychol. Rev. 84, 191–215. doi: 10.1037//0033-295x.84.2.191, 847061

[ref8] Booth J. Moseley G. L. Schiltenwolf M. Cashin A. Davies M. Hübscher M. (2017). Exercise for chronic musculoskeletal pain: a biopsychosocial approach. Musculoskelet. Care 15, 413–421. doi: 10.1002/msc.1191, 28371175

[ref9] Brophy R. H. Fillingham Y. A. (2022). AAOS clinical practice guideline summary: management of osteoarthritis of the knee (nonarthroplasty), third edition. J. Am. Acad. Orthop. Surg. 30, e721–e729. doi: 10.5435/jaaos-d-21-01233, 35383651

[ref10] Burns L. C. Ritvo S. E. Ferguson M. K. Clarke H. Seltzer Z. Katz J. (2015). Pain catastrophizing as a risk factor for chronic pain after total knee arthroplasty: a systematic review. J. Pain Res. 8, 21–32. doi: 10.2147/jpr.S64730, 25609995 PMC4294690

[ref11] Cai L. Liu Y. Xu H. Xu Q. Wang Y. Lyu P. (2018). Incidence and risk factors of kinesiophobia after total knee arthroplasty in Zhengzhou, China: a cross-sectional study. J. Arthroplast. 33, 2858–2862. doi: 10.1016/j.arth.2018.04.028, 29776855

[ref12] Chou R. Gordon D. B. de Leon-Casasola O. A. Rosenberg J. M. Bickler S. Brennan T. . (2016). Management of postoperative pain: a clinical practice guideline from the American pain society, the American Society of Regional Anesthesia and Pain Medicine, and the American Society of Anesthesiologists' committee on regional anesthesia, executive committee, and administrative council. J. Pain 17, 131–157. doi: 10.1016/j.jpain.2015.12.008, 26827847

[ref13] Dahlberg K. Jaensson M. Nilsson U. Eriksson M. Odencrants S. (2018). Holding it together-patients' perspectives on postoperative recovery when using an e-assessed follow-up: qualitative study. JMIR Mhealth Uhealth 6:e10387. doi: 10.2196/10387, 29802094 PMC5993971

[ref14] Dupuis F. Cherif A. Batcho C. Massé-Alarie H. Roy J. S. (2023). The Tampa scale of Kinesiophobia: a systematic review of its psychometric properties in people with musculoskeletal pain. Clin. J. Pain 39, 236–247. doi: 10.1097/ajp.0000000000001104, 36917768

[ref15] Elo S. Kyngäs H. (2008). The qualitative content analysis process. J. Adv. Nurs. 62, 107–115. doi: 10.1111/j.1365-2648.2007.04569.x, 18352969

[ref16] Elwyn G. Frosch D. Thomson R. Joseph-Williams N. Lloyd A. Kinnersley P. . (2012). Shared decision making: a model for clinical practice. J. Gen. Intern. Med. 27, 1361–1367. doi: 10.1007/s11606-012-2077-6, 22618581 PMC3445676

[ref17] Filardo G. Merli G. Roffi A. Marcacci T. Berti Ceroni F. Raboni D. . (2017). Kinesiophobia and depression affect total knee arthroplasty outcome in a multivariate analysis of psychological and physical factors on 200 patients. Knee Surg. Sports Traumatol. Arthrosc. 25, 3417–3423. doi: 10.1007/s00167-016-4201-3, 27329175

[ref18] Filardo G. Roffi A. Merli G. Marcacci T. Ceroni F. B. Raboni D. . (2016). Patient kinesiophobia affects both recovery time and final outcome after total knee arthroplasty. Knee Surg. Sports Traumatol. Arthrosc. 24, 3322–3328. doi: 10.1007/s00167-015-3898-8, 26685685

[ref19] Fu X. She Y. Jin G. Liu C. Liu Z. Li W. . (2024). Comparison of robotic-assisted total knee arthroplasty: an updated systematic review and meta-analysis. J. Robot. Surg. 18:292. doi: 10.1007/s11701-024-02045-y, 39052153 PMC11272701

[ref20] Gilbey H. J. Ackland T. R. Wang A. W. Morton A. R. Trouchet T. Tapper J. (2003). Exercise improves early functional recovery after total hip arthroplasty. Clin. Orthop. Relat. Res. 408, 193–200. doi: 10.1097/00003086-200303000-00025, 12616059

[ref21] Goudie S. Dixon D. McMillan G. Ring D. McQueen M. (2018). Is use of a psychological workbook associated with improved disabilities of the arm, shoulder and hand scores in patients with distal radius fracture? Clin. Orthop. Relat. Res. 476, 832–845. doi: 10.1007/s11999.0000000000000095, 29406451 PMC6260104

[ref22] Hamilton D. F. Lane J. V. Gaston P. Patton J. T. Macdonald D. Simpson A. H. . (2013). What determines patient satisfaction with surgery? A prospective cohort study of 4709 patients following total joint replacement. BMJ Open 3:e002525. doi: 10.1136/bmjopen-2012-002525, 23575998 PMC3641464

[ref23] Hennink M. M. Kaiser B. N. Weber M. B. (2019). What influences saturation? Estimating sample sizes in focus group research. Qual. Health Res. 29, 1483–1496. doi: 10.1177/1049732318821692, 30628545 PMC6635912

[ref24] Hladkowicz E. Auais M. Kidd G. McIsaac D. I. Miller J. (2023). "I can't imagine having to do it on your own": a qualitative study on postoperative transitions in care from the perspectives of older adults with frailty. BMC Geriatr. 23:848. doi: 10.1186/s12877-023-04576-9, 38093180 PMC10716948

[ref25] Jourdan C. Poiraudeau S. Descamps S. Nizard R. Hamadouche M. Anract P. . (2012). Comparison of patient and surgeon expectations of total hip arthroplasty. PLoS One 7:e30195. doi: 10.1371/journal.pone.0030195, 22272303 PMC3260245

[ref26] Kaambwa B. Lancsar E. McCaffrey N. Chen G. Gill L. Cameron I. D. . (2015). Investigating consumers' and informal carers' views and preferences for consumer directed care: a discrete choice experiment. Soc. Sci. Med. 140, 81–94. doi: 10.1016/j.socscimed.2015.06.034, 26210656

[ref27] Kneck Å. Audulv Å. (2019). Analyzing variations in changes over time: development of the pattern-oriented longitudinal analysis approach. Nurs. Inq. 26:e12288. doi: 10.1111/nin.12288, 30834658

[ref28] Knoop J. de Joo J. W. Brandt H. Dekker J. Ostelo R. (2022). Patients' and clinicians' experiences with stratified exercise therapy in knee osteoarthritis: a qualitative study. BMC Musculoskelet. Disord. 23:559. doi: 10.1186/s12891-022-05496-2, 35681162 PMC9178540

[ref29] Larsson F. Strömbäck U. Rysst Gustafsson S. Engström Å. (2022). Postoperative recovery: experiences of patients who have undergone orthopedic day surgery. J. Perianesth. Nurs. 37, 515–520. doi: 10.1016/j.jopan.2021.10.012, 35279387

[ref30] Lihong Z. Y. (2023). A qualitative study on the supportive care needs of liver transplant recipients. Chin. J. Nurs. 58, 2741–2746.

[ref31] Londhe S. B. Shah R. V. Agrawal P. O. Pestonji J. M. Londhe S. S. Langaliya M. K. (2021a). Education, engagement and provision of empathy by trained counselor enhances the patient satisfaction after total knee arthroplasty. J. Clin. Orthop. Trauma 17, 191–194. doi: 10.1016/j.jcot.2021.03.011, 33898238 PMC8047450

[ref32] Londhe S. B. Shah R. V. Patwardhan M. Doshi A. P. Londhe S. S. Subhedar K. (2021b). Understanding the apprehension and concern haunting patients before a total knee arthroplasty. Art 3:14. doi: 10.1186/s42836-021-00069-5, 35236475 PMC8796401

[ref33] Luque-Suarez A. Martinez-Calderon J. Falla D. (2019). Role of kinesiophobia on pain, disability and quality of life in people suffering from chronic musculoskeletal pain: a systematic review. Br. J. Sports Med. 53, 554–559. doi: 10.1136/bjsports-2017-098673, 29666064

[ref34] Mancuso C. A. Graziano S. Briskie L. M. Peterson M. G. Pellicci P. M. Salvati E. A. . (2008). Randomized trials to modify patients' preoperative expectations of hip and knee arthroplasties. Clin. Orthop. Relat. Res. 466, 424–431. doi: 10.1007/s11999-007-0052-z, 18196427 PMC2505138

[ref35] Min L. Mazzurco L. Gure T. R. Cigolle C. T. Lee P. Bloem C. . (2015). Longitudinal functional recovery after geriatric cardiac surgery. J. Surg. Res. 194, 25–33. doi: 10.1016/j.jss.2014.10.043, 25483736 PMC4346442

[ref36] Minetto M. A. Quilico E. Massazza F. Oprandi G. Busso C. Gasparini G. . (2025). Innovative digital approaches to characterize core factors of patients with late-stage knee osteoarthritis: a cross-sectional study. Front. Digit. Health 7:1709182. doi: 10.3389/fdgth.2025.1709182 41608161 PMC12835352

[ref37] Murray D. W. Parkinson R. W. (2018). Usage of unicompartmental knee arthroplasty. Bone Joint J 100-B, 432–435. doi: 10.1302/0301-620x.100b4.Bjj-2017-0716.R1, 29629577

[ref38] Nambi G. Basuodan R. M. Alwhaibi R. M. Ebrahim E. E. Verma A. Syed S. . (2023). Clinical and endocrinological responses to different exercise training methods in chronic low back pain: a randomized controlled trial. Endocr. Metab. Immune Disord. Drug Targets 23, 801–810. doi: 10.2174/1871530323666221031151721, 36321229

[ref39] Nuevo M. Rodríguez-Rodríguez D. Jauregui R. Fabrellas N. Zabalegui A. Conti M. . (2024). Telerehabilitation following fast-track total knee arthroplasty is effective and safe: a randomized controlled trial with the ReHub® platform. Disabil. Rehabil. 46, 2629–2639. doi: 10.1080/09638288.2023.2228689, 37403684

[ref40] Ottenvall Hammar I. Dahlin-Ivanoff S. Wilhelmson K. Eklund K. (2014). Shifting between self-governing and being governed: a qualitative study of older persons' self-determination. BMC Geriatr. 14:126. doi: 10.1186/1471-2318-14-126, 25432268 PMC4280698

[ref41] Padovan A. M. Kuvačić G. Gulotta F. Sellami M. Bruno C. Isoardi M. . (2018). A new integrative approach to increase quality of life by reducing pain and fear of movement in patients undergoing total hip arthroplasty: the IARA model. Psychol. Health Med. 23, 1223–1230. doi: 10.1080/13548506.2018.1488080, 29944000

[ref42] Partridge J. S. Harari D. Dhesi J. K. (2012). Frailty in the older surgical patient: a review. Age Ageing 41, 142–147. doi: 10.1093/ageing/afr182, 22345294

[ref43] Pendlimari R. Holubar S. D. Hassinger J. P. Cima R. R. (2012). Assessment of colon cancer literacy in screening colonoscopy patients: a validation study. J. Surg. Res. 175, 221–226. doi: 10.1016/j.jss.2011.04.036, 21737097

[ref44] Phelps E. E. Tutton E. Griffin X. Baird J. (2019). A qualitative study of patients' experience of recovery after a distal femoral fracture. Injury 50, 1750–1755. doi: 10.1016/j.injury.2019.07.021, 31371167

[ref45] Pinto D. Danilovich M. K. Hansen P. Finn D. J. Chang R. W. Holl J. L. . (2017). Qualitative development of a discrete choice experiment for physical activity interventions to improve knee osteoarthritis. Arch. Phys. Med. Rehabil. 98, 1210–1216.e1. doi: 10.1016/j.apmr.2016.11.024, 28034720 PMC13095379

[ref46] Ramkumar P. N. Haeberle H. S. Ramanathan D. Cantrell W. A. Navarro S. M. Mont M. A. . (2019). Remote patient monitoring using mobile health for total knee arthroplasty: validation of a wearable and machine learning-based surveillance platform. J. Arthroplast. 34, 2253–2259. doi: 10.1016/j.arth.2019.05.021, 31128890

[ref47] Riddle D. L. Wade J. B. Jiranek W. A. Kong X. (2010). Preoperative pain catastrophizing predicts pain outcome after knee arthroplasty. Clin. Orthop. Relat. Res. 468, 798–806. doi: 10.1007/s11999-009-0963-y, 19585177 PMC2816776

[ref48] Shin K. S. Lee E. H. (2018). Relationships of health literacy to self-care behaviors in people with diabetes aged 60 and above: empowerment as a mediator. J. Adv. Nurs. 74, 2363–2372. doi: 10.1111/jan.13738, 29893030

[ref49] Šťastný E. Trč T. Philippou T. (2016). Rehabilitation after total knee and hip arthroplasty [Rehabilitace po totální náhradě kyčelního a kolenního kloubu]. Cas. Lek. Cesk. 155, 427–432. 28098473

[ref50] Suc J. Prokosch H. U. Ganslandt T. (2009). Applicability of Lewin s change management model in a hospital setting. Methods Inf. Med. 48, 419–428. doi: 10.3414/me9235, 19696950

[ref51] Swinkels-Meewisse E. J. Swinkels R. A. Verbeek A. L. Vlaeyen J. W. Oostendorp R. A. (2003). Psychometric properties of the Tampa scale for kinesiophobia and the fear-avoidance beliefs questionnaire in acute low back pain. Man. Ther. 8, 29–36. doi: 10.1054/math.2002.0484, 12586559

[ref52] Tretter F. Löffler-Stastka H. (2024). Cognitive dissonance and mindset perturbations during crisis: "eco-socio-psycho-somatic" perspectives. World J. Psychiatry 14, 215–224. doi: 10.5498/wjp.v14.i2.215, 38464764 PMC10921281

[ref53] Trocoli T. O. Botelho R. V. (2016). Prevalence of anxiety, depression and kinesiophobia in patients with low back pain and their association with the symptoms of low back spinal pain. Rev. Bras. Reumatol. 56, 330–336. doi: 10.1016/j.rbre.2016.02.010, 27476626

[ref54] Wong W. S. Chow Y. F. Chen P. P. Wong S. Fielding R. (2015). A longitudinal analysis on pain treatment satisfaction among Chinese patients with chronic pain: predictors and association with medical adherence, disability, and quality of life. Qual. Life Res. 24, 2087–2097. doi: 10.1007/s11136-015-0955-1, 25749925

[ref55] Xie M. Yin L. Guo Y. Zhang X. Zhao R. (2024). Current status and influencing factors of kinesiophobia in patients with peritoneal dialysis: a multicenter cross-sectional study. BMC Nephrol. 25:404. doi: 10.1186/s12882-024-03851-0, 39529009 PMC11555836

[ref56] Xu Huiping Z. Y. Yanjin L. Yuru G. Libo C. Haojie Z. Yali N. . (2021). Chinese journal of nursing: a study on factors influencing fear of movement in patients after total knee arthroplasty. Chin. J. Nurs. 56, 1460–1465.

[ref57] Yanyan C. L. (2019). A qualitative study on early postoperative functional exercise experiences among patients with motion phobia undergoing total knee arthroplasty. Chin. J. Nurs. 54, 1663–1668.

[ref58] Zelle D. M. Corpeleijn E. Klaassen G. Schutte E. Navis G. Bakker S. J. (2016). Fear of movement and low self-efficacy are important barriers in physical activity after renal transplantation. PLoS One 11:e0147609. doi: 10.1371/journal.pone.0147609, 26844883 PMC4742485

[ref59] Zhu J. Weng H. Ou P. Li L. (2023). Use and acceptance of smart elderly care apps among Chinese medical staff and older individuals: web-based hybrid survey study. JMIR Form. Res. 7:e41919. doi: 10.2196/41919, 37310777 PMC10337394

[ref60] Ziyan W. Zhang L. X. Yu Q. Zheng M. Lu B. Chen J. . (2022). A study on the trajectory of anxiety levels in patients following percutaneous coronary intervention. Chin. J. Nurs. 57, 1035–1041.

